# Supplementary material for: Spatiotemporal identification of druggable binding sites using deep learning
Source: Commun Biol. 2020 Oct 27;3:618. doi: 10.1038/s42003-020-01350-0 (PMC7591901; doi:10.1038/s42003-020-01350-0)
Supplement: Supplementary file 1 — Supplementary Information [file 42003_2020_1350_MOESM1_ESM.pdf]

Supplementary Information for
Spatiotemporal identification of druggable binding sites using deep learning

Igor Kozlovskii[1] and Petr Popov[1]

[1]Skolkovo Institute of Science and Technology, Moscow, Russia

September 25, 2020

Binding site definition

P2Rank binding site filtering criterion

This criterion filters small molecules with less than 4 atoms, as well as HOH, DOD, WAT, NAG, MAN, UNK, GLC, ADA, MPD, GOL, SO4, PO4 molecules. The ligand must be within 4Å of a protein, and the distance from the ligand center to protein must be at least 5.5Å. A prediction is considered as true positive, if the minimum distance from the predicted coordinates to the ligand is less or equal than 4Å.

BiteNet binding site filtering criterion

This criterion filters out small molecules with less than 14 number of atoms, as well as detergent and buffer molecules (see Supplementary Table 1). Then binding site interface is defined as the number of protein atoms in the proximity of 4Åfrom the ligand, and binding sites with the interface less than 20 atoms are filtered out. A prediction is considered as true positive, if the minimum distance between the predicted coordinates and the ligand is less or equal than 4Å.

BiteNet binding site filtering criterion for training

This criterion differs from the previous one only in the true positive definition. Namely, a prediction is considered as true positive, if the minimum distance between the predicted coordinates and the geometric center of the binding site is less or equal than 4Å.

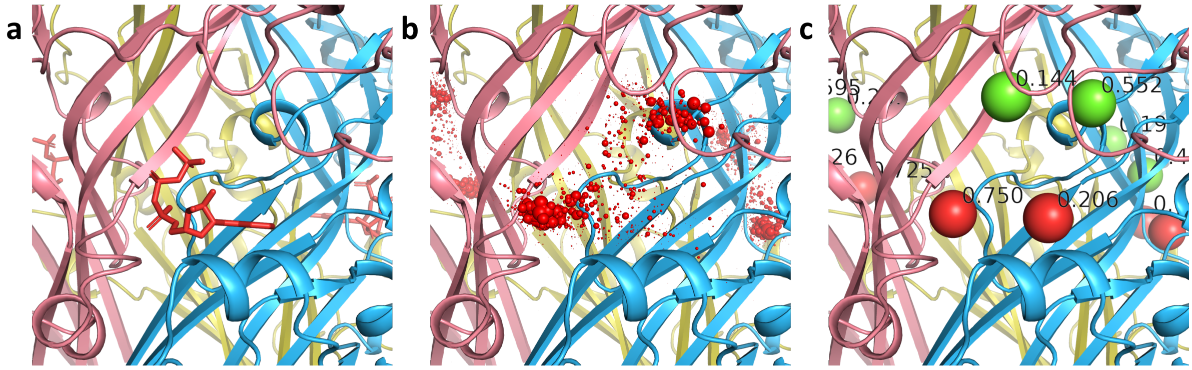

Supplementary Figure 1: BiteNet predictions for the ATP-bound P2X3 receptor trimer augmented with 50 replicas. (**a**) the ATP-binding site, ATP is shown with red sticks) (**b**) BiteNet predictions with no non max suppression. (**c**) BiteNet predictions filtered by the non max suppression with $distance\_threshold =$ 8Å.

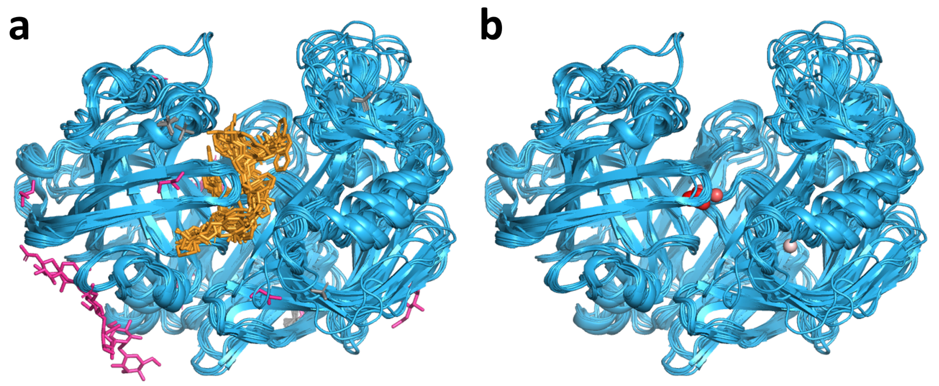

Supplementary Figure 2: Small molecules and BiteNet predictions for 29 proteins of the IPR001461 family from the HOLO4K dataset. **a** Small molecules corresponding to the relevant binding sites according to both BiteNet and P2Rank filtering criteria (yellow sticks), and only to the P2Rank filtering criterion (magenta sticks). **b** BiteNet predictions are shown with spheres colored from white to red with respect to the probability score.

Supplementary Table 1: List of the PDB identifiers filtered on the dataset preprocessing stage

Type	Pdb code
unknown ligands	UNL
detergents	12P, 15P, 1PE, 2CV, 2PE, 78M, 78N, BCR, BNG, BOG, BTB, C14, C8E, CDL, CLR, CM5, CPS, DAO, DGA, DGD, DMU, DXC, L2P, LDA, LFA, LHG, LI1, LMG, LMT, LMU, MPG, MQ7, MTN, MYR, MYS, NS5, OLA, OLB, OLC, P4C, P6G, PC1, PCW, PE4, PE5, PE8, PEE, PG6, PGV, PL9, PLC, OLM, PTY, PX4, SPN, SPO, SQD, STE, TGL, U10, YO1, HOH
modified residues	CSD, HYP, BMT, 5HP, ABA, AIB, CSW, OCS, DAL, DAR, DSG, DSP, DCY, CRO, DGL, DGN, DHI, DIL, DIV, DLE, DLY, DPN, DPR, DSN, DTH, DTR, DTY, DVA, CGU, KCX, LLP, CXM, FME, MLE, MVA, NLE, PTR, ORN, SEP, TPO, PCA, PVL, SAR, CEA, CSO, CSS, CSX, CME, TYS, TPQ, STY, NH2, CBX, ACE, FOR, IVA, BOC, LYR, MSE
cofactors	ADP, AMP, ATP, CMP, COA, FAD, FMN, NAP, NDP
carbohydrates	BGC, GLC, MAN, BMA, FUC, GAL, GLA, NAG, NGA, SIA, XYS

Source: `https://www.globalphasing.com/buster/manual/maketnt/manual/lib_val/library_validation.html`

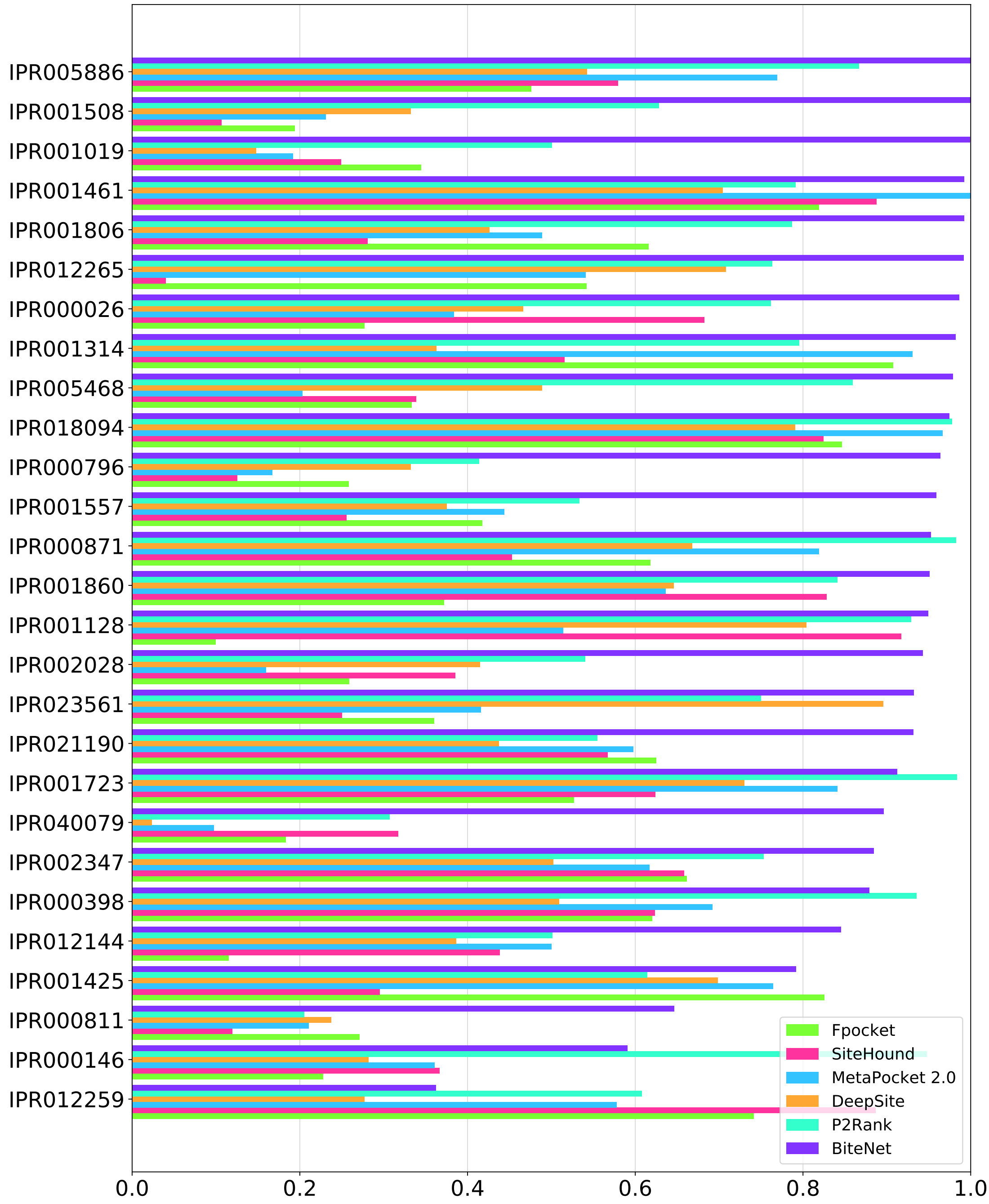

Supplementary Figure 3: The average precision metric for different methods with respect to the most represented protein families in the HOLO4K benchmark.

Supplementary Table 2: Performance of the deep learning models trained with different parameters.

Voxel size, Å	Density cutoff, Å	Cube size	Cell size	Stride	γ	λ	Minibatch	Average precision
1.0	2.0	48	4	32	1e-05	5	64	0.307
1.0	2.0	48	8	32	1e-05	5	64	0.394
1.0	2.0	48	16	32	1e-05	5	64	0.353
1.0	2.0	64	16	32	1e-05	5	32	0.383
1.0	2.0	64	4	32	1e-05	5	32	0.314
1.0	2.0	64	8	32	1e-05	5	4	0.387
1.0	2.0	64	8	32	1e-05	5	8	0.331
1.0	2.0	64	8	32	1e-05	5	16	0.429
1.0	2.0	64	8	32	1e-05	5	32	0.404
1.0	1.0	80	16	32	1e-05	5	16	0.365
1.0	2.0	80	16	32	1e-05	5	16	0.343
1.0	4.0	80	16	32	1e-05	5	16	0.387
1.0	8.0	80	16	32	1e-05	5	16	0.355
1.0	16.0	80	16	32	1e-05	5	16	0.368
1.0	2.0	80	16	32	0	5	16	0.372
1.0	2.0	80	16	32	1e-01	5	16	0.216
1.0	2.0	80	16	32	1e-02	5	16	0.264
1.0	2.0	80	16	32	1e-03	5	16	0.299
1.0	2.0	80	16	32	1e-04	5	16	0.362
1.0	2.0	80	16	32	1e-05	5	16	0.371
1.0	2.0	80	16	32	1e-06	5	16	0.351
1.0	2.0	80	16	32	1e-05	1	16	0.368
1.0	2.0	80	16	32	1e-05	10	16	0.377
1.0	2.0	80	16	32	1e-05	100	16	0.333
1.0	2.0	80	16	32	1e-05	1000	16	0.346
1.0	2.0	80	16	16	1e-05	5	16	0.272
1.0	2.0	80	16	24	1e-05	5	16	0.354
1.0	2.0	80	16	32	1e-05	5	16	0.399
1.0	2.0	80	16	48	1e-05	5	16	0.400
1.0	2.0	80	4	32	1e-05	5	16	0.302
1.0	2.0	80	8	32	1e-05	5	16	0.387
0.8	1.6	64	8	32	1e-05	5	32	0.435
1.5	3.0	64	8	32	1e-05	5	32	0.332
2.0	4.0	64	8	32	1e-05	5	32	0.283
3.0	6.0	64	8	32	1e-05	5	32	0.147

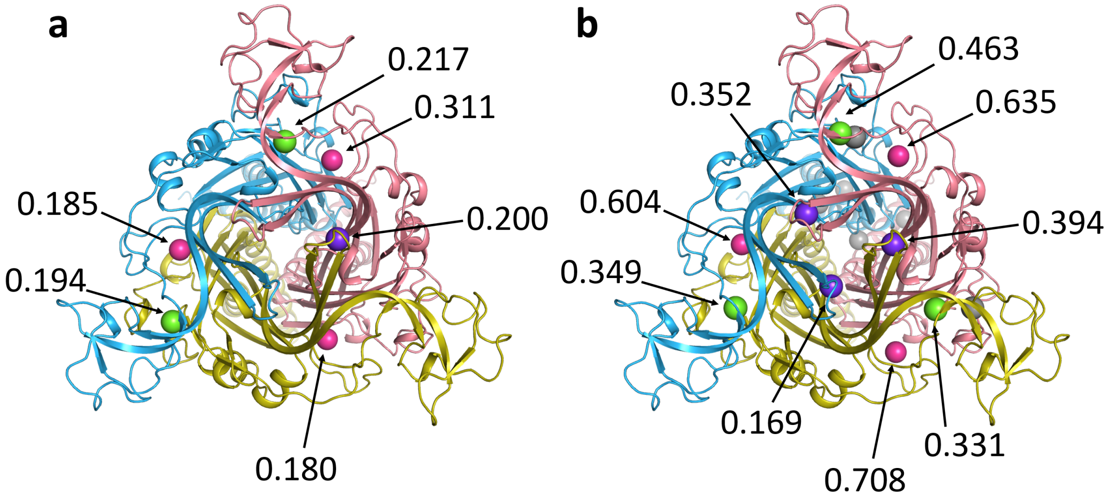

Supplementary Figure 4: BiteNet predictions obtained for the P2X3 trimer with no (**a**) or with (**b**) rotational replicas.

Supplementary Table 3: Performance metrics on the COACH420 subset of 239 proteins, which are not in the BiteNet training set, outputs of all methods are available for them and each structure contains at least one relevant binding site according to the P2Rank binding site filtering criterion.

Criterion	Method	Top-1			Top-3			Top-n			Top-(n+2)			All		
		prec	rec	ap	prec	rec	ap	prec	rec	ap	prec	rec	ap	prec	rec	ap
	Fpocket (239)	0.67	0.50	0.40	0.28	0.63	0.46	0.51	0.56	0.43	0.26	0.66	0.46	0.07	0.76	0.48
P2Rank	SiteHound (239)	0.58	0.44	0.29	0.29	0.65	0.33	0.47	0.54	0.33	0.26	0.68	0.34	0.02	**0.94**	0.34
binding	MetaPocket 2.0 (239)	0.70	0.53	0.41	0.29	0.67	0.48	0.52	0.58	0.44	0.27	0.69	0.48	0.16	0.74	0.49
site	DeepSite (239)	0.65	0.47	0.38	0.31	0.57	0.40	0.53	0.51	0.40	0.30	0.57	0.40	**0.25**	0.57	0.40
filtering	P2Rank (239)	0.83	0.63	0.54	0.33	0.70	0.54	0.65	0.68	0.57	0.31	0.72	0.54	0.15	0.75	0.54
criterion	**BiteNet** (239)	**0.87**	**0.67**	**0.64**	**0.38**	0.77	0.69	**0.69**	0.73	0.68	**0.36**	0.79	0.70	0.24	0.81	0.71
	**BiteNet R$^+$** (239)	**0.87**	0.66	0.62	0.34	**0.78**	**0.69**	0.67	**0.75**	**0.69**	0.31	**0.80**	**0.70**	0.10	0.86	**0.71**
	Fpocket (239)	0.49	0.67	0.37	0.20	0.80	0.41	0.67	0.68	0.54	0.21	0.80	0.41	0.05	0.93	0.42
BiteNet	SiteHound (239)	0.43	0.58	0.31	0.20	0.81	0.34	0.61	0.62	0.44	0.22	0.83	0.35	0.01	**0.99**	0.33
binding	MetaPocket 2.0 (239)	0.52	0.71	0.41	0.21	0.88	0.48	0.70	0.71	0.57	0.23	0.88	0.48	0.11	0.92	0.48
site	DeepSite (239)	0.48	0.64	0.41	0.22	0.75	0.42	0.65	0.64	0.51	0.24	0.75	0.42	**0.18**	0.75	0.42
filtering	P2Rank (239)	0.60	0.81	0.49	0.23	0.88	0.49	0.82	0.83	0.68	0.25	0.88	0.49	0.10	0.91	0.49
criterion	**BiteNet** (239)	0.62	0.84	0.57	**0.25**	0.92	0.59	**0.85**	0.85	**0.81**	**0.28**	0.92	0.59	0.16	0.94	0.60
	**BiteNet R$^+$** (239)	**0.62**	**0.85**	**0.63**	0.23	**0.93**	**0.66**	**0.85**	**0.86**	0.80	0.25	**0.94**	**0.67**	0.06	0.99	**0.67**
	Fpocket (239)	0.26	0.35	0.09	0.11	0.42	0.10	0.36	0.36	0.13	0.12	0.42	0.10	0.03	0.50	0.11
	SiteHound (239)	0.30	0.40	0.15	0.15	0.59	0.18	0.42	0.42	0.21	0.16	0.59	0.18	0.01	0.79	0.18
BiteNet	MetaPocket 2.0 (239)	0.29	0.40	0.12	0.12	0.51	0.15	0.39	0.40	0.17	0.14	0.51	0.15	0.07	0.60	0.16
criterion	DeepSite (239)	0.27	0.36	0.14	0.12	0.41	0.15	0.36	0.36	0.17	0.13	0.41	0.15	0.10	0.41	0.15
for training	P2Rank (239)	0.42	0.56	0.24	0.16	0.59	0.23	0.57	0.57	0.33	0.17	0.59	0.23	0.07	0.60	0.23
	**BiteNet** (239)	**0.54**	**0.73**	0.45	**0.22**	0.79	0.46	**0.73**	**0.74**	**0.63**	**0.24**	0.79	0.46	**0.14**	0.81	0.46
	**BiteNet R$^+$** (239)	0.52	0.72	**0.49**	0.19	**0.80**	**0.52**	0.72	0.73	0.62	0.21	**0.80**	**0.53**	0.05	**0.86**	**0.53**
		TP	FP	FN	TP	FP	FN	TP	FP	FN	TP	FP	FN	TP	FP	FN
	Fpocket (239)	159	80	174	229	488	128	178	167	155	237	575	119	312	3262	84
P2Rank	SiteHound (239)	139	100	196	242	475	124	165	184	162	254	573	113	**499**	14136	**22**
binding	MetaPocket 2.0 (239)	167	72	163	230	487	116	182	164	146	239	577	109	296	1217	90
site	DeepSite (239)	155	82	184	208	411	151	172	144	171	209	438	150	212	**549**	149
filtering	P2Rank (239)	197	40	131	236	435	104	215	113	111	243	500	97	262	1330	88
criterion	BiteNet (239)	**209**	**30**	**117**	275	**358**	80	231	**98**	95	281	**401**	75	307	742	68
	BiteNet R$^+$ (239)	**209**	**30**	119	**297**	418	**78**	**239**	110	**88**	**307**	515	**70**	407	2449	49
	Fpocket (239)	117	122	59	162	555	36	119	58	57	163	492	36	215	3359	13
BiteNet	SiteHound (239)	103	136	74	169	548	34	108	69	67	173	482	31	**323**	14312	**1**
binding	MetaPocket 2.0 (239)	123	116	52	166	551	22	124	53	51	166	489	22	209	1304	14
site	DeepSite (239)	113	124	63	145	474	44	115	61	63	145	432	44	147	**614**	44
filtering	P2Rank (239)	142	95	33	164	507	22	144	31	31	166	456	21	179	1413	16
criterion	BiteNet (239)	147	92	29	183	**450**	15	**151**	**26**	26	183	**397**	15	200	849	10
	BiteNet R$^+$ (239)	**148**	**91**	**27**	**199**	516	**12**	**151**	**26**	**25**	**200**	453	**11**	280	2576	2
	Fpocket (239)	62	177	115	75	642	102	63	114	114	75	580	102	89	3485	88
	SiteHound (239)	71	168	106	104	613	73	75	102	102	105	550	72	139	14496	38
BiteNet	MetaPocket 2.0 (239)	69	170	107	90	627	87	69	108	107	90	565	87	109	1404	71
criterion	DeepSite (239)	64	173	113	75	544	104	64	112	113	75	502	104	75	**686**	104
for training	P2Rank (239)	99	138	78	105	566	73	100	75	77	105	517	73	107	1485	71
	BiteNet (239)	**129**	**110**	**47**	**139**	**494**	37	**130**	**47**	**46**	139	**441**	37	143	906	33
	BiteNet R$^+$ (239)	125	114	50	**139**	576	**36**	127	50	48	**140**	513	**35**	**150**	2706	**25**

The best score values are denoted with bold font.

Supplementary Table 4: Performance metrics on the HOLO4K subset of 1682 proteins, which are not in the BiteNet training set, outputs of all methods are available for them and each structure contains at least one relevant binding site according to the P2Rank binding site filtering criterion.

Criterion	Method	Top-1			Top-3			Top-n			Top-(n+2)			All		
		prec	rec	ap	prec	rec	ap	prec	rec	ap	prec	rec	ap	prec	rec	ap
P2Rank binding site filtering criterion	Fpocket (1682)	0.68	0.38	0.28	0.37	0.62	0.41	0.54	0.59	0.40	0.32	0.70	0.44	0.07	0.81	0.45
	SiteHound (1682)	0.61	0.36	0.22	0.34	0.59	0.31	0.49	0.56	0.32	0.31	0.70	0.34	0.02	**0.97**	0.36
	MetaPocket 2.0 (1682)	0.67	0.39	0.25	0.35	0.62	0.38	0.53	0.59	0.37	0.31	0.69	0.41	0.20	0.73	0.42
	DeepSite (1682)	0.73	0.42	0.32	0.42	0.58	0.40	0.65	0.56	0.42	0.40	0.59	0.40	**0.35**	0.59	0.40
	P2Rank (1682)	0.85	0.50	0.41	0.43	0.73	0.58	0.65	0.73	0.60	0.36	0.78	0.60	0.14	0.81	0.61
	**BiteNet** (1682)	0.92	0.51	0.49	**0.53**	0.77	0.72	**0.78**	0.78	0.75	**0.46**	0.82	0.76	0.32	0.84	0.77
	**BiteNet R$^+$** (1682)	**0.93**	**0.51**	**0.50**	0.47	**0.78**	**0.74**	0.75	**0.80**	**0.77**	0.40	**0.85**	**0.79**	0.13	0.89	**0.79**
BiteNet binding site filtering criterion	Fpocket (1682)	0.45	0.45	0.21	0.24	0.71	0.31	0.60	0.62	0.41	0.26	0.79	0.34	0.04	0.89	0.34
	SiteHound (1682)	0.42	0.42	0.19	0.24	0.70	0.25	0.56	0.59	0.35	0.26	0.77	0.29	0.01	**1.00**	0.28
	MetaPocket 2.0 (1682)	0.48	0.48	0.21	0.24	0.73	0.32	0.61	0.64	0.40	0.26	0.79	0.33	0.13	0.84	0.34
	DeepSite (1682)	0.51	0.50	0.27	0.28	0.67	0.31	0.70	0.64	0.48	0.30	0.68	0.32	**0.24**	0.68	0.31
	P2Rank (1682)	0.60	0.60	0.35	0.29	0.85	0.48	0.79	0.83	0.68	0.30	0.89	0.51	0.09	0.92	0.50
	**BiteNet** (1682)	0.64	0.63	0.45	**0.36**	0.91	0.64	0.89	0.90	0.86	**0.37**	0.96	0.68	0.21	0.97	0.68
	**BiteNet R$^+$** (1682)	**0.65**	**0.63**	**0.48**	0.32	**0.91**	**0.68**	**0.89**	**0.91**	**0.88**	0.33	**0.96**	**0.71**	0.08	0.99	**0.71**
BiteNet criterion for training	Fpocket (1682)	0.27	0.26	0.06	0.14	0.41	0.10	0.35	0.35	0.12	0.16	0.45	0.11	0.03	0.53	0.11
	SiteHound (1682)	0.30	0.28	0.08	0.17	0.49	0.12	0.40	0.40	0.15	0.19	0.54	0.13	0.01	0.80	0.14
	MetaPocket 2.0 (1682)	0.27	0.26	0.06	0.14	0.40	0.09	0.34	0.34	0.10	0.15	0.45	0.10	0.08	0.50	0.10
	DeepSite (1682)	0.31	0.30	0.10	0.16	0.38	0.10	0.42	0.37	0.16	0.17	0.39	0.11	0.14	0.39	0.11
	P2Rank (1682)	0.41	0.39	0.14	0.19	0.54	0.17	0.52	0.52	0.25	0.20	0.56	0.18	0.06	0.58	0.18
	**BiteNet** (1682)	0.58	0.55	0.37	**0.32**	0.79	0.52	0.78	0.78	0.69	**0.33**	0.83	0.55	**0.19**	0.84	0.54
	**BiteNet R$^+$** (1682)	**0.59**	**0.56**	**0.40**	0.28	**0.81**	**0.57**	**0.80**	**0.80**	**0.72**	0.30	**0.85**	**0.59**	0.07	**0.88**	**0.59**
		TP	FP	FN	TP	FP	FN	TP	FP	FN	TP	FP	FN	TP	FP	FN
P2Rank binding site filtering criterion	Fpocket (1682)	1140	542	1997	2014	3022	1228	1788	1433	1342	2322	4236	971	3156	32013	630
	SiteHound (1682)	1022	660	2085	2023	3023	1315	1700	1538	1432	2440	4162	988	**4934**	132214	**113**
	MetaPocket 2.0 (1682)	1123	559	1980	1939	3107	1227	1719	1402	1344	2227	4107	1007	2591	8174	878
	DeepSite (1682)	1233	448	1875	1813	2328	1347	1653	869	1419	1829	2467	1331	1839	**3129**	1327
	P2Rank (1682)	1424	258	1616	2151	2696	871	2080	1078	886	2325	3912	714	2603	13944	604
	BiteNet (1682)	1551	131	1582	2503	**1868**	761	2461	**581**	698	2811	**2612**	569	3012	4974	521
	BiteNet R$^+$ (1682)	**1565**	**117**	**1573**	**2631**	2357	**729**	**2566**	660	**655**	**3043**	3463	**495**	4032	17646	358
BiteNet binding site filtering criterion	Fpocket (1682)	760	922	967	1313	3723	505	1058	698	664	1463	3652	378	2023	33146	187
	SiteHound (1682)	711	971	1017	1383	3663	532	1014	742	725	1554	3566	397	**3140**	134008	**1**
	MetaPocket 2.0 (1682)	801	881	917	1313	3733	471	1079	667	625	1444	3626	378	1709	9056	290
	DeepSite (1682)	854	827	874	1219	2922	575	1084	463	640	1226	2674	568	1234	**3734**	565
	P2Rank (1682)	1006	676	698	1463	3384	265	1388	365	307	1549	3417	196	1724	14823	141
	BiteNet (1682)	1082	600	652	1680	**2691**	165	1570	179	170	1837	**2659**	74	1987	5999	53
	BiteNet R$^+$ (1682)	**1095**	**587**	**644**	**1747**	3241	**158**	**1587**	**169**	**162**	**1932**	3150	**70**	2656	19022	16
BiteNet criterion for training	Fpocket (1682)	447	1235	1313	723	4313	1037	611	1152	1149	796	4326	964	938	34231	830
	SiteHound (1682)	499	1183	1262	863	4183	900	699	1064	1061	958	4169	804	1405	135743	360
	MetaPocket 2.0 (1682)	454	1228	1303	708	4338	1050	588	1165	1169	784	4293	977	894	9871	884
	DeepSite (1682)	519	1162	1241	700	3441	1089	644	909	1118	705	3198	1084	707	**4261**	1082
	P2Rank (1682)	681	1001	1077	938	3909	820	908	852	849	980	3991	777	1021	15526	737
	BiteNet (1682)	967	715	793	1384	**2987**	376	1373	382	387	1463	**3035**	297	1480	6506	280
	BiteNet R$^+$ (1682)	**985**	**697**	**774**	**1417**	3571	**342**	**1402**	**361**	**357**	**1499**	3590	**260**	**1552**	20126	**207**

The best score values are denoted with bold font.

Supplementary Table 5: Performance metrics for the entire COACH420 dataset.

Criterion	Method	Top-1			Top-3			Top-n			Top-(n+2)			All		
		prec	rec	ap	prec	rec	ap	prec	rec	ap	prec	rec	ap	prec	rec	ap
P2Rank binding site filtering criterion	Fpocket (420)	0.63	0.49	0.37	0.27	0.63	0.43	0.51	0.56	0.41	0.25	0.66	0.44	0.07	0.76	0.45
	SiteHound (284)	0.57	0.44	0.23	0.28	0.64	0.33	0.47	0.53	0.33	0.25	0.67	0.33	0.02	**0.93**	0.34
	MetaPocket 2.0 (417)	0.71	0.56	0.44	0.29	0.69	0.51	0.55	0.61	0.47	0.27	0.71	0.51	0.15	0.76	0.52
	DeepSite (420)	0.64	0.49	0.37	0.30	0.57	0.37	0.54	0.52	0.39	0.29	0.58	0.38	**0.25**	0.58	0.38
	P2Rank (420)	0.79	0.62	0.54	0.32	0.71	0.55	0.64	0.67	0.56	0.31	0.72	0.56	0.15	0.74	0.55
	**BiteNet** (420)	**0.83**	**0.65**	0.60	**0.37**	0.77	0.67	**0.70**	0.72	0.66	**0.36**	0.79	0.68	0.23	0.81	0.68
	**BiteNet** $\mathbf{R^+}$ (420)	0.83	0.65	**0.61**	0.34	**0.78**	**0.69**	0.66	**0.73**	**0.67**	0.31	**0.81**	**0.70**	0.09	0.85	**0.71**
BiteNet binding site filtering criterion	Fpocket (420)	0.48	0.66	0.37	0.19	0.79	0.40	0.67	0.67	0.50	0.21	0.79	0.40	0.05	0.91	0.41
	SiteHound (284)	0.44	0.59	0.31	0.20	0.81	0.34	0.61	0.62	0.43	0.22	0.83	0.35	0.01	**1.00**	0.34
	MetaPocket 2.0 (417)	0.52	0.73	0.41	0.21	0.88	0.47	0.72	0.73	0.58	0.23	0.88	0.47	0.11	0.93	0.48
	DeepSite (420)	0.47	0.64	0.37	0.21	0.73	0.36	0.65	0.64	0.47	0.23	0.73	0.36	**0.18**	0.73	0.36
	P2Rank (420)	0.59	0.81	0.51	0.23	0.89	0.52	0.82	0.82	0.71	0.25	0.90	0.52	0.11	0.92	0.52
	**BiteNet** (420)	**0.60**	0.83	0.55	**0.25**	0.92	0.58	**0.84**	0.84	0.78	**0.28**	0.92	0.58	0.15	0.94	0.58
	**BiteNet** $\mathbf{R^+}$ (420)	0.60	**0.83**	**0.59**	0.23	**0.94**	**0.63**	0.83	**0.84**	**0.79**	0.25	**0.95**	**0.64**	0.06	0.99	**0.64**
BiteNet criterion for training	Fpocket (420)	0.27	0.37	0.11	0.11	0.46	0.12	0.37	0.37	0.14	0.12	0.46	0.12	0.03	0.53	0.12
	SiteHound (284)	0.30	0.40	0.14	0.15	0.59	0.17	0.42	0.42	0.20	0.16	0.59	0.17	0.010	0.79	0.17
	MetaPocket 2.0 (417)	0.27	0.38	0.10	0.12	0.48	0.12	0.38	0.38	0.14	0.13	0.48	0.12	0.06	0.55	0.13
	DeepSite (420)	0.25	0.34	0.11	0.11	0.39	0.12	0.34	0.34	0.14	0.12	0.39	0.12	0.10	0.39	0.12
	P2Rank (420)	0.38	0.52	0.20	0.15	0.56	0.20	0.53	0.52	0.27	0.16	0.56	0.20	0.07	0.57	0.20
	**BiteNet** (420)	0.51	0.70	0.42	**0.21**	0.77	0.44	**0.70**	0.70	0.59	**0.23**	0.77	0.44	**0.13**	0.79	0.44
	**BiteNet** $\mathbf{R^+}$ (420)	**0.51**	**0.71**	**0.46**	0.20	**0.82**	**0.50**	0.71	**0.72**	**0.62**	0.22	**0.82**	**0.51**	0.05	**0.86**	**0.51**
		TP	FP	FN	TP	FP	FN	TP	FP	FN	TP	FP	FN	TP	FP	FN
P2Rank binding site filtering criterion	Fpocket (420)	265	155	298	386	869	214	301	279	260	403	999	197	522	5612	143
	SiteHound (284)	161	123	225	278	574	144	191	210	188	290	679	133	577	16527	27
	MetaPocket 2.0 (417)	294	123	255	405	846	178	322	256	226	423	979	166	517	2117	139
	DeepSite (420)	268	149	300	358	727	250	295	240	282	360	769	248	366	**961**	247
	P2Rank (420)	329	87	220	405	755	171	353	194	195	414	846	162	445	2186	152
	BiteNet (420)	**348**	**71**	**204**	469	**635**	134	387	**163**	163	480	**696**	124	528	1364	113
	BiteNet R^+ (420)	**348**	72	207	**510**	744	**126**	**393**	188	**160**	**528**	881	**111**	**696**	4249	**85**
BiteNet binding site filtering criterion	Fpocket (420)	200	220	104	272	983	65	203	102	101	273	868	65	360	5774	28
	SiteHound (284)	124	160	88	204	648	40	129	83	81	208	572	37	391	16713	1
	MetaPocket 2.0 (417)	217	200	83	291	960	35	219	84	81	291	846	35	363	2271	22
	DeepSite (420)	194	223	110	247	838	84	196	107	110	247	758	84	253	**1074**	82
	P2Rank (420)	245	171	57	290	870	33	247	54	55	292	780	32	315	2316	26
	BiteNet (420)	**251**	**168**	53	320	**784**	26	**255**	**49**	50	321	**692**	25	359	1533	17
	BiteNet R^+ (420)	**251**	169	**52**	**347**	907	**18**	**255**	50	**49**	**350**	789	**15**	**488**	4457	**4**
BiteNet criterion for training	Fpocket (420)	113	307	192	139	1116	166	114	191	191	139	1002	166	163	5971	142
	SiteHound (284)	84	200	128	124	728	88	88	124	124	125	655	87	168	16936	45
	MetaPocket 2.0 (417)	114	303	188	146	1105	157	115	188	187	146	991	157	173	2461	136
	DeepSite (420)	104	313	201	125	960	186	104	199	201	125	880	186	127	**1200**	185
	P2Rank (420)	157	259	148	171	989	135	158	143	147	171	901	135	176	2455	130
	BiteNet (420)	212	**207**	92	235	**869**	69	213	91	91	235	**778**	69	241	1651	63
	BiteNet R^+ (420)	**213**	**207**	**90**	**247**	1007	**56**	**216**	**89**	**87**	**249**	890	**54**	**261**	4684	**42**

The best score values are denoted with bold font. Top TP, FP, FN scores apply only to methods assessed on the same set of proteins as BiteNet.

Supplementary Table 6: Performance metrics for the entire HOLO4K dataset.

Criterion	Method	Top-1			Top-3			Top-n			Top-(n+2)			All		
		prec	rec	ap	prec	rec	ap	prec	rec	ap	prec	rec	ap	prec	rec	ap
P2Rank binding site filtering criterion	Fpocket (4009)	0.62	0.29	0.19	0.37	0.51	0.31	0.48	0.53	0.33	0.32	0.63	0.36	0.06	0.78	0.38
	SiteHound (2878)	0.56	0.27	0.14	0.34	0.49	0.22	0.44	0.51	0.24	0.30	0.64	0.26	0.02	**0.97**	0.29
	MetaPocket 2.0 (2575)	0.65	0.38	0.23	0.35	0.62	0.37	0.53	0.59	0.36	0.31	0.69	0.40	0.20	0.73	0.41
	DeepSite (3991)	0.69	0.33	0.23	0.43	0.48	0.30	0.62	0.47	0.32	0.42	0.49	0.31	**0.37**	0.49	0.31
	P2Rank (4542)	0.81	0.40	0.31	0.46	0.65	0.50	0.61	0.69	0.54	0.37	0.75	0.56	0.13	0.79	0.56
	**BiteNet** (4542)	0.91	**0.42**	0.40	**0.57**	0.70	0.66	**0.76**	0.79	0.75	**0.48**	0.84	0.76	0.29	0.86	0.77
	**BiteNet R$^+$** (4542)	**0.92**	0.42	**0.40**	0.52	**0.71**	**0.67**	0.75	**0.81**	**0.77**	0.44	**0.86**	**0.78**	0.14	0.90	**0.79**
BiteNet binding site filtering criterion	Fpocket (4009)	0.41	0.33	0.14	0.25	0.58	0.23	0.51	0.54	0.32	0.26	0.68	0.26	0.04	0.86	0.28
	SiteHound (2878)	0.39	0.32	0.12	0.23	0.56	0.17	0.48	0.51	0.23	0.26	0.68	0.21	0.01	**1.00**	0.22
	MetaPocket 2.0 (2575)	0.48	0.46	0.20	0.25	0.73	0.31	0.61	0.64	0.39	0.27	0.79	0.34	0.14	0.84	0.34
	DeepSite (3991)	0.48	0.38	0.19	0.29	0.55	0.24	0.65	0.52	0.35	0.31	0.55	0.25	**0.25**	0.56	0.24
	P2Rank (4542)	0.58	0.47	0.27	0.31	0.74	0.41	0.73	0.77	0.60	0.32	0.83	0.47	0.09	0.87	0.45
	**BiteNet** (4542)	0.64	0.50	0.36	**0.39**	0.82	0.58	0.85	0.87	0.82	**0.40**	0.94	0.67	0.19	0.97	0.66
	**BiteNet R$^+$** (4542)	**0.64**	**0.50**	**0.38**	0.35	**0.82**	**0.62**	**0.86**	**0.88**	**0.84**	0.37	**0.95**	**0.71**	0.09	0.99	**0.70**
BiteNet criterion for training	Fpocket (4009)	0.23	0.17	0.04	0.13	0.31	0.06	0.28	0.28	0.08	0.14	0.36	0.07	0.02	0.46	0.08
	SiteHound (2878)	0.26	0.20	0.05	0.16	0.38	0.07	0.32	0.32	0.09	0.17	0.45	0.09	0.01	0.77	0.10
	MetaPocket 2.0 (2575)	0.26	0.24	0.05	0.14	0.39	0.08	0.33	0.33	0.10	0.16	0.44	0.09	0.08	0.49	0.10
	DeepSite (3991)	0.27	0.21	0.06	0.16	0.29	0.07	0.35	0.28	0.10	0.17	0.29	0.07	0.14	0.29	0.07
	P2Rank (4542)	0.36	0.28	0.08	0.19	0.43	0.12	0.44	0.44	0.17	0.19	0.48	0.14	0.05	0.51	0.13
	**BiteNet** (4542)	0.56	0.44	0.29	**0.34**	0.71	0.47	0.74	0.74	0.65	**0.35**	0.80	0.53	**0.17**	0.83	0.53
	**BiteNet R$^+$** (4542)	**0.57**	**0.44**	**0.32**	0.32	**0.73**	**0.52**	**0.76**	**0.76**	**0.69**	0.33	**0.82**	**0.59**	0.08	**0.86**	**0.58**
		TP	FP	FN	TP	FP	FN	TP	FP	FN	TP	FP	FN	TP	FP	FN
P2Rank binding site filtering criterion	Fpocket (4009)	2490	1519	6771	4788	7208	4612	4656	4818	4496	6006	11435	3492	8905	99369	2052
	SiteHound (2878)	1608	1270	4900	3366	5268	3418	3159	3577	3282	4511	7981	2437	10637	276793	181
	MetaPocket 2.0 (2575)	1677	898	3114	2948	4777	1913	2637	2227	2075	3424	6359	1552	4009	12422	1344
	DeepSite (3991)	2760	1228	6379	4250	5306	4905	3961	2374	5015	4324	5639	4823	4346	6791	4814
	P2Rank (4542)	3699	842	6475	6199	7001	3796	6553	4044	3280	7311	11702	2710	8321	48776	2220
	BiteNet (4542)	4128	414	**6254**	7400	**4790**	3212	8182	**2118**	2240	9368	**7855**	1741	10492	**18811**	1457
	BiteNet R$^+$ (4542)	**4160**	**382**	6269	**7759**	5763	**3152**	**8490**	2194	**2066**	**9999**	9583	**1549**	**13145**	51252	**1071**
BiteNet binding site filtering criterion	Fpocket (4009)	1656	2353	3504	3168	8828	2168	2722	2499	2420	3730	9488	1666	5783	102491	745
	SiteHound (2878)	1126	1752	2532	2312	6322	1624	1832	1874	1820	2821	6641	1182	6928	280502	3
	MetaPocket 2.0 (2575)	1231	1344	1525	2081	5644	773	1717	1082	1022	2328	5568	595	2775	13656	454
	DeepSite (3991)	1918	2070	3216	2872	6684	2367	2616	1400	2482	2916	6159	2321	2935	8202	2314
	P2Rank (4542)	2630	1911	3121	4252	8948	1514	4308	1586	1373	4826	9801	992	5464	51633	742
	BiteNet (4542)	2892	1650	**2939**	5029	**7161**	1067	5078	794	757	6007	**7595**	348	6894	**22409**	171
	BiteNet R$^+$ (4542)	**2904**	**1638**	2943	**5233**	8289	**1042**	**5148**	**748**	**704**	**6272**	8632	**314**	**8653**	55744	**84**
BiteNet criterion for training	Fpocket (4009)	906	3103	4321	1608	10388	3616	1444	3789	3778	1876	11354	3346	2418	105856	2813
	SiteHound (2878)	748	2130	2961	1398	7236	2316	1193	2521	2514	1662	7808	2049	2858	284572	867
	MetaPocket 2.0 (2575)	677	1898	2140	1101	6624	1719	914	1896	1902	1241	6666	1580	1415	15016	1436
	DeepSite (3991)	1086	2902	4126	1554	8002	3706	1431	2595	3782	1571	7508	3689	1577	9560	3685
	P2Rank (4542)	1622	2919	4285	2507	10693	3393	2589	3318	3304	2832	11805	3062	2998	54099	2902
	BiteNet (4542)	2566	1976	3341	4158	**8032**	1744	4369	1515	1529	4735	**8873**	1163	4900	**24403**	998
	BiteNet R$^+$ (4542)	**2599**	**1943**	**3308**	**4281**	9241	**1621**	**4493**	**1416**	**1407**	**4850**	10067	**1050**	**5065**	59332	**835**

The best score values are denoted with bold font. Top TP, FP, FN scores apply only to methods assessed on the same set of proteins as BiteNet.

Supplementary Table 7: Performance metrics on the COACH420 subset of 352 proteins, which are not in the BiteNet training set and each structure contains at least one relevant binding site according to the P2Rank binding site filtering criterion.

Criterion	Method	Top-1			Top-3			Top-n			Top-(n+2)			All		
		prec	rec	ap	prec	rec	ap	prec	rec	ap	prec	rec	ap	prec	rec	ap
P2Rank binding site filtering criterion	Fpocket (352)	0.66	0.50	0.37	0.28	0.64	0.43	0.51	0.56	0.41	0.26	0.66	0.44	0.07	0.76	0.45
	SiteHound (240)	0.58	0.44	0.29	0.28	0.64	0.33	0.47	0.53	0.33	0.26	0.67	0.34	0.02	**0.94**	0.34
	MetaPocket 2.0 (349)	0.73	0.56	0.44	0.30	0.69	0.51	0.55	0.61	0.48	0.27	0.71	0.52	0.16	0.76	0.53
	DeepSite (352)	0.65	0.47	0.37	0.31	0.56	0.38	0.53	0.50	0.38	0.30	0.56	0.38	**0.26**	0.57	0.38
	P2Rank (352)	0.81	0.62	0.53	0.33	0.70	0.54	0.64	0.67	0.55	0.31	0.72	0.54	0.15	0.74	0.54
	**BiteNet** (352)	0.86	**0.65**	0.61	**0.38**	0.77	0.68	**0.69**	0.72	0.66	**0.36**	0.79	0.69	0.23	0.81	0.69
	**BiteNet R$^+$** (352)	**0.86**	0.65	**0.61**	0.34	**0.78**	**0.69**	0.66	**0.73**	**0.67**	0.31	**0.81**	**0.70**	0.09	0.85	**0.71**
BiteNet binding site filtering criterion	Fpocket (352)	0.48	0.67	0.36	0.19	0.79	0.39	0.68	0.69	0.52	0.21	0.79	0.39	0.04	0.92	0.40
	SiteHound (240)	0.43	0.58	0.31	0.20	0.80	0.33	0.61	0.62	0.44	0.22	0.82	0.34	0.01	**0.99**	0.33
	MetaPocket 2.0 (349)	0.51	0.73	0.40	0.21	0.88	0.46	0.72	0.73	0.59	0.23	0.88	0.46	0.10	0.92	0.47
	DeepSite (352)	0.45	0.63	0.37	0.21	0.73	0.36	0.64	0.63	0.48	0.22	0.73	0.37	**0.17**	0.73	0.36
	P2Rank (352)	0.58	0.82	0.48	0.22	0.89	0.48	0.82	0.83	0.70	0.25	0.89	0.48	0.10	0.92	0.48
	**BiteNet** (352)	0.59	0.82	0.52	**0.25**	0.91	0.56	0.83	0.83	0.78	**0.27**	0.92	0.56	0.15	0.95	0.56
	**BiteNet R$^+$** (352)	**0.59**	**0.83**	**0.57**	0.22	**0.93**	**0.61**	**0.83**	**0.84**	**0.79**	0.25	**0.94**	**0.61**	0.06	0.98	**0.62**
BiteNet criterion for training	Fpocket (352)	0.26	0.36	0.09	0.11	0.44	0.10	0.37	0.37	0.13	0.12	0.44	0.10	0.02	0.52	0.11
	SiteHound (240)	0.30	0.40	0.14	0.14	0.58	0.17	0.42	0.42	0.21	0.16	0.59	0.18	0.01	0.79	0.18
	MetaPocket 2.0 (349)	0.28	0.40	0.11	0.12	0.50	0.13	0.40	0.40	0.16	0.13	0.50	0.13	0.06	0.57	0.14
	DeepSite (352)	0.25	0.35	0.13	0.11	0.40	0.13	0.35	0.35	0.16	0.12	0.40	0.13	0.10	0.40	0.13
	P2Rank (352)	0.38	0.53	0.20	0.15	0.57	0.20	0.54	0.53	0.29	0.16	0.57	0.20	0.06	0.59	0.20
	**BiteNet** (352)	0.50	0.70	0.40	**0.21**	0.78	0.43	0.70	0.71	0.59	**0.23**	0.78	0.43	**0.13**	0.81	0.43
	**BiteNet R$^+$** (352)	**0.50**	**0.71**	**0.45**	0.19	**0.80**	**0.48**	**0.71**	**0.72**	**0.62**	0.21	**0.81**	**0.48**	0.05	**0.85**	**0.48**
		TP	FP	FN	TP	FP	FN	TP	FP	FN	TP	FP	FN	TP	FP	FN
P2Rank binding site filtering criterion	Fpocket (352)	231	121	256	337	717	185	262	241	223	352	840	170	457	4879	121
	SiteHound (240)	139	101	197	242	478	125	165	185	163	254	576	114	501	14310	22
	MetaPocket 2.0 (349)	254	95	223	348	699	158	278	223	197	365	824	146	448	1759	121
	DeepSite (352)	228	122	268	305	604	222	250	211	252	306	642	221	311	**791**	220
	P2Rank (352)	285	65	192	349	635	151	307	169	169	358	721	142	388	1938	132
	BiteNet (352)	301	51	**176**	407	**527**	117	334	**146**	141	418	**592**	107	458	1156	97
	BiteNet R$^+$ (352)	**303**	**49**	177	**438**	612	**113**	**341**	163	**137**	**454**	743	**98**	**590**	3598	**75**
BiteNet binding site filtering criterion	Fpocket (352)	169	183	82	229	825	52	172	80	79	230	724	52	303	5033	20
	SiteHound (240)	103	137	75	169	551	35	108	70	68	173	485	32	325	14486	1
	MetaPocket 2.0 (349)	179	170	68	237	810	31	180	70	67	237	711	31	297	1910	20
	DeepSite (352)	158	192	93	202	707	69	160	91	93	202	639	69	206	**896**	68
	P2Rank (352)	203	147	46	238	746	28	205	45	44	240	665	27	262	2064	21
	BiteNet (352)	206	146	45	266	**668**	22	210	42	42	267	**589**	21	297	1317	13
	BiteNet R$^+$ (352)	**208**	**144**	**42**	**283**	767	**17**	**211**	**41**	**40**	**286**	664	**14**	**393**	3795	**4**
BiteNet criterion for training	Fpocket (352)	91	261	161	111	943	141	92	160	160	111	843	141	132	5204	120
	SiteHound (240)	71	169	107	104	616	74	75	103	103	105	553	73	140	14671	38
	MetaPocket 2.0 (349)	99	250	150	124	923	126	99	151	150	124	824	126	147	2060	107
	DeepSite (352)	89	261	163	106	803	151	89	162	163	106	735	151	108	**994**	150
	P2Rank (352)	133	217	119	144	840	109	134	116	118	144	761	109	149	2177	104
	BiteNet (352)	176	176	75	196	**738**	55	177	75	74	196	**660**	55	202	1412	49
	BiteNet R$^+$ (352)	**177**	**175**	**73**	**200**	850	**50**	**179**	**73**	**71**	**202**	748	**48**	**212**	3976	**38**

The best score values are denoted with bold font. Top TP, FP, FN scores apply only to methods assessed on the same set of proteins as BiteNet.

Supplementary Table 8: Performance metrics on the HOLO4K subset of 4169 proteins, which are not in the BiteNet training set and each structure contains at least one relevant binding site according to the P2Rank binding site filtering criterion.

Criterion	Method	Top-1			Top-3			Top-n			Top-(n+2)			All		
		prec	rec	ap	prec	rec	ap	prec	rec	ap	prec	rec	ap	prec	rec	ap
P2Rank binding site filtering criterion	Fpocket (3687)	0.62	0.29	0.19	0.37	0.51	0.31	0.47	0.52	0.33	0.31	0.63	0.36	0.06	0.78	0.38
	SiteHound (2655)	0.56	0.28	0.15	0.33	0.50	0.22	0.43	0.52	0.24	0.30	0.64	0.26	0.02	**0.97**	0.29
	MetaPocket 2.0 (2359)	0.65	0.38	0.23	0.35	0.62	0.37	0.52	0.58	0.36	0.31	0.69	0.40	0.20	0.73	0.41
	DeepSite (3669)	0.70	0.33	0.24	0.43	0.48	0.31	0.62	0.47	0.33	0.41	0.49	0.31	**0.37**	0.49	0.31
	P2Rank (4169)	0.82	0.40	0.31	0.46	0.65	0.50	0.61	0.69	0.54	0.37	0.75	0.56	0.13	0.79	0.56
	**BiteNet** (4169)	0.91	**0.42**	0.41	**0.57**	0.70	0.66	**0.76**	0.79	0.75	**0.48**	0.84	0.77	0.29	0.86	0.77
	**BiteNet R$^+$** (4169)	**0.92**	0.42	**0.41**	0.52	**0.71**	**0.67**	0.75	**0.81**	**0.77**	0.43	**0.86**	**0.78**	0.14	0.90	**0.79**
BiteNet binding site filtering criterion	Fpocket (3687)	0.40	0.34	0.14	0.24	0.59	0.22	0.51	0.53	0.31	0.25	0.68	0.25	0.04	0.85	0.26
	SiteHound (2655)	0.38	0.33	0.12	0.23	0.57	0.17	0.48	0.51	0.24	0.25	0.69	0.21	0.01	**1.00**	0.22
	MetaPocket 2.0 (2359)	0.47	0.47	0.20	0.24	0.73	0.30	0.61	0.64	0.40	0.26	0.79	0.33	0.13	0.84	0.33
	DeepSite (3669)	0.47	0.39	0.19	0.28	0.55	0.24	0.66	0.53	0.36	0.30	0.56	0.25	**0.24**	0.56	0.24
	P2Rank (4169)	0.56	0.48	0.26	0.30	0.75	0.40	0.73	0.77	0.61	0.31	0.83	0.46	0.08	0.88	0.44
	**BiteNet** (4169)	0.62	**0.51**	0.36	**0.37**	0.82	0.57	**0.85**	0.87	0.82	**0.39**	0.94	0.65	0.18	0.97	0.64
	**BiteNet R$^+$** (4169)	**0.62**	0.51	**0.38**	0.34	**0.83**	**0.60**	0.86	**0.88**	**0.85**	0.35	**0.95**	**0.69**	0.08	0.99	**0.68**
BiteNet criterion for training	Fpocket (3687)	0.22	0.18	0.04	0.13	0.31	0.06	0.28	0.28	0.08	0.14	0.36	0.07	0.02	0.47	0.07
	SiteHound (2655)	0.26	0.21	0.05	0.16	0.39	0.08	0.33	0.33	0.10	0.17	0.46	0.09	0.01	0.78	0.10
	MetaPocket 2.0 (2359)	0.26	0.25	0.05	0.14	0.40	0.08	0.33	0.33	0.10	0.15	0.44	0.09	0.08	0.50	0.10
	DeepSite (3669)	0.28	0.22	0.06	0.16	0.30	0.07	0.37	0.29	0.11	0.17	0.30	0.07	0.14	0.30	0.07
	P2Rank (4169)	0.36	0.29	0.08	0.19	0.44	0.12	0.45	0.45	0.18	0.19	0.49	0.14	0.05	0.52	0.13
	**BiteNet** (4169)	0.55	0.45	0.29	**0.33**	0.71	0.46	0.75	0.75	0.65	**0.34**	0.81	0.52	**0.16**	0.84	0.51
	**BiteNet R$^+$** (4169)	**0.56**	**0.45**	**0.31**	0.30	**0.73**	**0.51**	**0.76**	**0.77**	**0.69**	0.32	**0.83**	**0.57**	0.08	**0.86**	**0.56**
		TP	FP	FN	TP	FP	FN	TP	FP	FN	TP	FP	FN	TP	FP	FN
P2Rank binding site filtering criterion	Fpocket (3687)	2297	1390	6156	4374	6661	4221	4215	4434	4144	5454	10520	3210	8082	90636	1905
	SiteHound (2655)	1492	1163	4445	3102	4863	3089	2870	3285	2985	4095	7370	2218	9601	252876	174
	MetaPocket 2.0 (2359)	1544	815	2824	2691	4386	1754	2387	2047	1907	3114	5828	1430	3653	11423	1235
	DeepSite (3669)	2570	1096	5766	3906	4892	4452	3633	2170	4558	3969	5216	4382	3988	6280	4374
	P2Rank (4169)	3410	758	5860	5663	6449	3452	5967	3686	2988	6658	10715	2466	7563	44345	2022
	BiteNet (4169)	3806	363	**5638**	6736	**4419**	2912	7439	**1950**	2040	8490	**7213**	1587	9473	**17044**	1334
	BiteNet R$^+$ (4169)	**3832**	**337**	5652	**7058**	5347	**2858**	**7718**	2026	**1875**	**9050**	8856	**1413**	**11854**	46832	**977**
BiteNet binding site filtering criterion	Fpocket (3687)	1480	2207	3049	2793	8242	1896	2367	2218	2147	3256	8682	1476	5056	93662	670
	SiteHound (2655)	1015	1640	2198	2069	5896	1393	1610	1648	1587	2490	6078	1020	6057	256420	2
	MetaPocket 2.0 (2359)	1104	1255	1320	1841	5236	674	1509	954	896	2047	5082	526	2445	12631	400
	DeepSite (3669)	1739	1927	2765	2561	6237	2040	2330	1205	2142	2594	5714	2005	2610	7658	1999
	P2Rank (4169)	2352	1816	2685	3753	8359	1301	3782	1380	1196	4235	8946	862	4777	47131	645
	BiteNet (4169)	2583	1586	**2517**	4417	**6738**	915	4451	697	655	5244	**6992**	302	5978	**20539**	151
	BiteNet R$^+$ (4169)	**2593**	**1576**	2522	**4585**	7820	**898**	**4510**	**662**	**604**	**5456**	7982	**280**	**7511**	51175	**69**
BiteNet criterion for training	Fpocket (3687)	811	2876	3779	1430	9605	3157	1270	3326	3315	1664	10285	2921	2139	96579	2454
	SiteHound (2655)	686	1969	2575	1275	6690	1991	1079	2187	2180	1503	7073	1760	2549	259928	724
	MetaPocket 2.0 (2359)	622	1737	1858	984	6093	1498	818	1655	1661	1102	6037	1381	1259	13817	1249
	DeepSite (3669)	1011	2655	3564	1420	7378	3202	1311	2233	3265	1431	6880	3191	1436	8832	3188
	P2Rank (4169)	1483	2685	3690	2251	9861	2916	2329	2845	2832	2542	10648	2620	2681	49227	2487
	BiteNet (4169)	2304	1865	2869	3675	**7480**	1493	3856	1303	1310	4174	**8067**	992	4315	**22202**	851
	BiteNet R$^+$ (4169)	**2319**	**1850**	**2854**	**3776**	8629	**1392**	**3952**	**1232**	**1214**	**4262**	9188	**904**	**4445**	54241	**721**

The best score values are denoted with bold font. Top TP, FP, FN scores apply only to methods assessed on the same set of proteins as BiteNet.

Supplementary Table 9: Performance metrics on the COACH420 subset of 344 proteins, which are not in both BiteNet training and validation set and each structure contains at least one relevant binding site according to the P2Rank binding site filtering criterion.

Criterion	Method	Top-1			Top-3			Top-n			Top-(n+2)			All		
		prec	rec	ap	prec	rec	ap	prec	rec	ap	prec	rec	ap	prec	rec	ap
P2Rank binding site filtering criterion	Fpocket (344)	0.65	0.49	0.37	0.28	0.64	0.43	0.51	0.56	0.41	0.26	0.67	0.44	0.07	0.76	0.45
	SiteHound (237)	0.57	0.43	0.28	0.28	0.64	0.32	0.46	0.53	0.32	0.26	0.67	0.33	0.02	**0.94**	0.33
	MetaPocket 2.0 (341)	0.72	0.56	0.44	0.30	0.69	0.51	0.55	0.61	0.48	0.27	0.71	0.52	0.16	0.76	0.53
	DeepSite (344)	0.66	0.48	0.38	0.31	0.57	0.39	0.54	0.51	0.39	0.30	0.57	0.39	**0.26**	0.57	0.39
	P2Rank (344)	0.82	0.62	0.53	0.33	0.70	0.54	0.64	0.67	0.55	0.31	0.72	0.55	0.15	0.74	0.54
	**BiteNet** (344)	0.85	**0.65**	0.61	**0.38**	0.77	0.68	**0.69**	0.72	0.66	**0.36**	0.79	0.69	0.23	0.81	0.69
	**BiteNet R$^+$** (344)	**0.86**	0.65	**0.61**	0.34	**0.78**	**0.69**	0.66	**0.73**	**0.67**	0.31	**0.80**	**0.70**	0.09	0.85	**0.71**
BiteNet binding site filtering criterion	Fpocket (344)	0.47	0.67	0.35	0.19	0.80	0.38	0.68	0.68	0.52	0.21	0.80	0.38	0.04	0.92	0.39
	SiteHound (237)	0.42	0.57	0.30	0.20	0.80	0.33	0.60	0.61	0.43	0.22	0.82	0.34	0.01	**0.99**	0.32
	MetaPocket 2.0 (341)	0.51	0.73	0.40	0.21	0.88	0.46	0.72	0.73	0.60	0.23	0.88	0.47	0.10	0.93	0.47
	DeepSite (344)	0.46	0.65	0.38	0.21	0.74	0.37	0.65	0.65	0.50	0.23	0.74	0.38	**0.18**	0.75	0.37
	P2Rank (344)	0.58	0.82	0.48	0.22	0.89	0.48	0.82	0.83	0.70	0.24	0.89	0.48	0.10	0.92	0.48
	**BiteNet** (344)	0.58	0.82	0.52	**0.24**	0.91	0.55	0.83	0.84	0.78	**0.27**	0.92	0.55	0.15	0.95	0.56
	**BiteNet R$^+$** (344)	**0.59**	**0.84**	**0.57**	0.22	**0.93**	**0.61**	**0.84**	**0.84**	**0.79**	0.25	**0.95**	**0.61**	0.06	0.99	**0.61**
BiteNet criterion for training	Fpocket (344)	0.25	0.35	0.08	0.10	0.43	0.10	0.35	0.35	0.12	0.11	0.43	0.10	0.02	0.51	0.10
	SiteHound (237)	0.29	0.39	0.13	0.14	0.58	0.17	0.41	0.41	0.20	0.16	0.58	0.17	0.01	0.78	0.17
	MetaPocket 2.0 (341)	0.28	0.39	0.11	0.12	0.49	0.13	0.39	0.39	0.16	0.13	0.49	0.13	0.06	0.57	0.14
	DeepSite (344)	0.26	0.36	0.13	0.12	0.41	0.14	0.37	0.36	0.17	0.13	0.41	0.14	0.10	0.42	0.14
	P2Rank (344)	0.37	0.52	0.20	0.14	0.56	0.20	0.53	0.53	0.29	0.16	0.56	0.20	0.06	0.58	0.20
	**BiteNet** (344)	0.49	0.70	0.40	**0.21**	0.78	0.42	0.70	0.70	0.59	**0.23**	0.78	0.42	**0.12**	0.80	0.43
	**BiteNet R$^+$** (344)	**0.50**	**0.71**	**0.45**	0.19	**0.80**	**0.48**	**0.71**	**0.72**	**0.62**	0.21	**0.81**	**0.48**	0.05	**0.85**	**0.49**
		TP	FP	FN	TP	FP	FN	TP	FP	FN	TP	FP	FN	TP	FP	FN
P2Rank binding site filtering criterion	Fpocket (344)	224	120	252	330	700	181	255	237	219	345	820	166	448	4773	119
	SiteHound (237)	136	101	197	239	472	125	162	185	163	251	570	114	498	14175	22
	MetaPocket 2.0 (341)	247	94	219	341	682	154	271	219	193	358	804	142	441	1719	117
	DeepSite (344)	227	115	258	302	584	214	248	203	243	303	622	213	307	**759**	212
	P2Rank (344)	279	63	188	342	619	148	301	164	165	351	704	139	381	1907	129
	BiteNet (344)	294	50	**172**	398	**514**	114	326	**143**	138	408	**578**	105	448	1127	95
	BiteNet R$^+$ (344)	**296**	**48**	173	**430**	596	**110**	**333**	160	**134**	**444**	726	**97**	**575**	3503	**74**
BiteNet binding site filtering criterion	Fpocket (344)	163	181	80	223	807	50	166	78	77	224	706	50	295	4926	20
	SiteHound (237)	100	137	75	166	545	35	105	70	68	170	479	32	322	14351	1
	MetaPocket 2.0 (341)	173	168	66	231	792	29	174	68	65	231	693	29	291	1869	18
	DeepSite (344)	157	185	86	200	686	63	159	84	86	200	618	63	203	**863**	62
	P2Rank (344)	197	145	44	231	730	27	199	43	42	233	649	26	255	2033	20
	BiteNet (344)	200	144	43	258	**654**	21	204	40	40	259	**575**	20	289	1286	12
	BiteNet R$^+$ (344)	**202**	**142**	**40**	**276**	750	**16**	**205**	**39**	**38**	**279**	647	**13**	**381**	3697	**3**
BiteNet criterion for training	Fpocket (344)	85	259	159	105	925	139	86	158	158	105	825	139	124	5097	120
	SiteHound (237)	68	169	107	101	610	74	72	103	103	102	547	73	137	14536	38
	MetaPocket 2.0 (341)	94	247	147	119	904	123	94	148	147	119	805	123	142	2018	104
	DeepSite (344)	89	253	155	106	780	143	89	154	155	106	712	143	108	**958**	142
	P2Rank (344)	128	214	116	138	823	107	129	113	115	138	744	107	143	2145	102
	BiteNet (344)	170	174	73	189	**723**	54	171	73	72	189	**645**	54	195	1380	48
	BiteNet R$^+$ (344)	**172**	**172**	**70**	**194**	832	**48**	**174**	**70**	**68**	**196**	730	**46**	**206**	3872	**36**

The best score values are denoted with bold font. Top TP, FP, FN scores apply only to methods assessed on the same set of proteins as BiteNet.

Supplementary Table 10: Performance metrics on the HOLO4K subset of 4121 proteins, which are not in BiteNet training and validation sets and each structure contains at least one relevant binding site according to the P2Rank binding site filtering criterion.

Criterion	Method	Top-1			Top-3			Top-n			Top-(n+2)			All		
		prec	rec	ap	prec	rec	ap	prec	rec	ap	prec	rec	ap	prec	rec	ap
	Fpocket (3649)	0.62	0.29	0.19	0.37	0.52	0.31	0.48	0.52	0.33	0.31	0.63	0.36	0.06	0.78	0.38
P2Rank	SiteHound (2628)	0.57	0.28	0.15	0.34	0.50	0.23	0.44	0.52	0.24	0.30	0.64	0.26	0.02	**0.97**	0.29
binding	MetaPocket 2.0 (2334)	0.66	0.38	0.24	0.35	0.62	0.37	0.53	0.58	0.36	0.31	0.69	0.40	0.20	0.73	0.41
site	DeepSite (3631)	0.70	0.33	0.24	0.43	0.49	0.31	0.62	0.48	0.33	0.42	0.50	0.32	**0.37**	0.50	0.32
filtering	P2Rank (4121)	0.82	0.40	0.32	0.46	0.65	0.50	0.61	0.70	0.55	0.37	0.75	0.56	0.13	0.80	0.57
criterion	**BiteNet** (4121)	0.91	**0.42**	0.41	**0.57**	0.70	0.66	**0.76**	0.79	0.75	**0.48**	0.84	0.77	0.29	0.87	0.77
	**BiteNet R**$^{+}$ (4121)	**0.92**	0.42	**0.41**	0.52	**0.71**	**0.67**	0.75	**0.81**	**0.77**	0.43	**0.86**	**0.78**	0.14	0.90	**0.79**
	Fpocket (3649)	0.40	0.34	0.14	0.23	0.59	0.22	0.51	0.53	0.32	0.25	0.68	0.25	0.04	0.86	0.26
BiteNet	SiteHound (2628)	0.38	0.33	0.12	0.23	0.58	0.17	0.49	0.52	0.24	0.25	0.69	0.21	0.01	**1.00**	0.22
binding	MetaPocket 2.0 (2334)	0.47	0.47	0.20	0.24	0.73	0.31	0.61	0.64	0.40	0.26	0.79	0.33	0.13	0.84	0.33
site	DeepSite (3631)	0.47	0.40	0.20	0.28	0.56	0.24	0.66	0.53	0.37	0.30	0.56	0.25	**0.24**	0.57	0.24
filtering	P2Rank (4121)	0.56	0.48	0.26	0.30	0.75	0.40	0.73	0.77	0.61	0.31	0.83	0.46	0.08	0.88	0.44
criterion	**BiteNet** (4121)	0.62	**0.51**	0.36	**0.37**	0.82	0.56	0.85	0.87	0.82	**0.38**	0.94	0.65	0.18	0.97	0.64
	**BiteNet R**$^{+}$ (4121)	**0.62**	0.51	**0.38**	0.33	**0.83**	**0.60**	**0.86**	**0.88**	**0.85**	0.35	**0.95**	**0.69**	0.08	0.99	**0.68**
	Fpocket (3649)	0.22	0.18	0.04	0.13	0.31	0.06	0.28	0.28	0.08	0.14	0.37	0.07	0.02	0.47	0.07
	SiteHound (2628)	0.26	0.21	0.05	0.16	0.40	0.08	0.33	0.34	0.10	0.18	0.47	0.09	0.01	0.78	0.10
BiteNet	MetaPocket 2.0 (2334)	0.26	0.25	0.06	0.14	0.40	0.09	0.33	0.33	0.11	0.15	0.45	0.10	0.08	0.50	0.10
criterion	DeepSite (3631)	0.28	0.22	0.06	0.16	0.30	0.07	0.37	0.29	0.11	0.17	0.31	0.07	0.14	0.31	0.07
for training	P2Rank (4121)	0.36	0.29	0.08	0.19	0.44	0.12	0.45	0.46	0.19	0.19	0.50	0.14	0.05	0.52	0.13
	**BiteNet** (4121)	0.55	0.45	0.29	**0.33**	0.71	0.46	0.75	0.75	0.65	**0.34**	0.81	0.52	**0.16**	0.84	0.51
	**BiteNet R**$^{+}$ (4121)	**0.55**	**0.45**	**0.31**	0.30	**0.73**	**0.50**	**0.76**	**0.77**	**0.69**	0.32	**0.83**	**0.57**	0.08	**0.86**	**0.56**
		TP	FP	FN	TP	FP	FN	TP	FP	FN	TP	FP	FN	TP	FP	FN
	Fpocket (3649)	2277	1372	6053	4332	6590	4142	4169	4355	4070	5399	10375	3144	7994	89297	1858
P2Rank	SiteHound (2628)	1486	1142	4354	3087	4797	3007	2849	3208	2913	4064	7249	2156	9478	249053	161
binding	MetaPocket 2.0 (2334)	1534	800	2788	2671	4331	1728	2371	2015	1879	3093	5752	1405	3630	11285	1211
site	DeepSite (3631)	2549	1079	5664	3874	4839	4361	3605	2140	4465	3937	5161	4291	3956	6218	4283
filtering	P2Rank (4121)	3375	745	5753	5596	6376	3383	5893	3616	2933	6577	10562	2415	7481	43588	1972
criterion	BiteNet (4121)	3759	362	**5542**	6642	**4375**	2857	7336	**1919**	2001	8373	**7118**	1551	9348	**16820**	1299
	BiteNet R^{+} (4121)	**3785**	**336**	5556	**6965**	5296	**2807**	**7619**	1988	**1834**	**8933**	8744	**1379**	**11714**	46205	**947**
	Fpocket (3649)	1461	2188	2996	2755	8167	1861	2333	2179	2110	3213	8577	1446	4984	92307	654
BiteNet	SiteHound (2628)	1009	1619	2151	2054	5830	1354	1599	1605	1546	2466	5994	988	5961	252570	2
binding	MetaPocket 2.0 (2334)	1094	1240	1290	1822	5180	653	1495	927	872	2027	5011	506	2423	12492	381
site	DeepSite (3631)	1719	1909	2712	2532	6181	1994	2306	1174	2093	2565	5658	1959	2581	7593	1953
filtering	P2Rank (4121)	2318	1802	2631	3689	8283	1277	3722	1353	1170	4166	8836	842	4706	46363	627
criterion	BiteNet (4121)	2539	1582	**2475**	4328	**6689**	908	4371	687	646	5145	**6913**	299	5871	**20297**	150
	BiteNet R^{+} (4121)	**2549**	**1572**	2478	**4499**	7762	**888**	**4433**	**649**	**593**	**5361**	7891	**274**	**7388**	50531	**69**
	Fpocket (3649)	800	2849	3717	1409	9513	3105	1250	3273	3262	1640	10161	2872	2106	95185	2414
	SiteHound (2628)	683	1945	2524	1271	6613	1941	1074	2138	2131	1495	6973	1714	2515	256016	704
BiteNet	MetaPocket 2.0 (2334)	617	1717	1822	973	6029	1468	811	1621	1627	1090	5958	1352	1245	13670	1222
criterion	DeepSite (3631)	1001	2627	3501	1406	7307	3143	1298	2191	3205	1417	6809	3132	1422	8752	3129
for training	P2Rank (4121)	1465	2655	3618	2216	9756	2861	2296	2791	2775	2503	10508	2568	2640	48429	2437
	BiteNet (4121)	2269	1852	2814	3611	**7406**	1467	3792	1277	1284	4105	**7958**	971	4244	**21924**	832
	BiteNet R^{+} (4121)	**2280**	**1841**	**2803**	**3707**	8554	**1371**	**3885**	**1209**	**1191**	**4187**	9077	**889**	**4366**	53553	**710**

The best score values are denoted with bold font. Top TP, FP, FN scores apply only to methods assessed on the same set of proteins as BiteNet.

Supplementary Table 11: Performance metrics on the COACH420 subset of 238 proteins, which are not in the BiteNet training set and each structure contains at least one relevant binding site according to the P2Rank binding site filtering criterion.

Criterion	Method	Top-1			Top-3			Top-n			Top-(n+2)			All		
		prec	rec	ap	prec	rec	ap	prec	rec	ap	prec	rec	ap	prec	rec	ap
P2Rank binding site filtering criterion	Fpocket (238)	0.72	0.61	0.47	0.29	0.74	0.52	0.64	0.65	0.50	0.28	0.75	0.53	0.07	0.86	0.54
	SiteHound (167)	0.62	0.56	0.40	0.29	0.76	0.43	0.55	0.60	0.41	0.27	0.77	0.43	0.02	**0.96**	0.44
	MetaPocket 2.0 (236)	0.79	0.68	0.58	0.32	0.82	0.66	0.68	0.70	0.59	0.30	0.82	0.66	0.16	0.86	0.67
	DeepSite (238)	0.68	0.58	0.46	0.31	0.68	0.48	0.61	0.60	0.47	0.31	0.68	0.48	**0.26**	0.68	0.47
	P2Rank (238)	0.87	0.76	0.67	0.33	0.83	0.67	0.75	0.78	0.68	0.32	0.84	0.67	0.15	0.86	0.67
	**BiteNet** (238)	0.89	0.77	**0.74**	**0.39**	0.87	0.80	**0.80**	**0.81**	**0.77**	**0.38**	0.89	0.81	0.24	0.91	0.81
	**BiteNet R**$^+$ (238)	**0.90**	**0.77**	0.73	0.34	**0.87**	**0.80**	0.78	0.81	0.77	0.33	**0.89**	**0.81**	0.10	0.94	**0.82**
BiteNet binding site filtering criterion	Fpocket (238)	0.71	0.67	0.51	0.28	0.79	0.55	0.68	0.69	0.52	0.27	0.79	0.55	0.06	0.92	0.58
	SiteHound (167)	0.62	0.58	0.41	0.28	0.80	0.44	0.61	0.62	0.44	0.28	0.82	0.45	0.02	**0.99**	0.44
	MetaPocket 2.0 (236)	0.76	0.73	0.58	0.31	0.88	0.67	0.72	0.73	0.59	0.30	0.88	0.67	0.15	0.92	0.68
	DeepSite (238)	0.66	0.63	0.48	0.30	0.73	0.49	0.64	0.63	0.48	0.30	0.73	0.49	**0.25**	0.73	0.49
	P2Rank (238)	0.86	0.82	0.70	0.33	0.89	0.69	0.82	0.83	0.70	0.32	0.89	0.69	0.15	0.92	0.69
	**BiteNet** (238)	0.87	0.82	0.77	**0.37**	0.91	0.82	0.83	0.83	0.78	**0.36**	0.92	0.82	0.23	0.95	0.84
	**BiteNet R**$^+$ (238)	**0.87**	**0.83**	**0.78**	0.33	**0.93**	**0.84**	**0.83**	**0.84**	**0.79**	0.33	**0.94**	**0.85**	0.09	0.98	**0.86**
BiteNet criterion for training	Fpocket (238)	0.38	0.36	0.13	0.16	0.44	0.15	0.37	0.37	0.13	0.15	0.44	0.15	0.04	0.52	0.16
	SiteHound (167)	0.43	0.40	0.19	0.21	0.58	0.23	0.42	0.42	0.21	0.21	0.59	0.23	0.01	0.79	0.23
	MetaPocket 2.0 (236)	0.42	0.40	0.16	0.17	0.50	0.19	0.40	0.40	0.16	0.17	0.50	0.19	0.10	0.57	0.20
	DeepSite (238)	0.37	0.35	0.17	0.17	0.40	0.18	0.35	0.35	0.16	0.17	0.40	0.18	0.14	0.40	0.18
	P2Rank (238)	0.56	0.53	0.29	0.21	0.57	0.29	0.54	0.53	0.29	0.21	0.57	0.29	0.09	0.59	0.29
	**BiteNet** (238)	0.74	0.70	0.60	**0.31**	0.78	0.63	0.70	0.71	0.59	**0.31**	0.78	0.63	**0.19**	0.81	0.64
	**BiteNet R**$^+$ (238)	**0.74**	**0.71**	**0.61**	0.28	**0.80**	**0.66**	**0.71**	**0.72**	**0.62**	0.28	**0.81**	**0.67**	0.08	**0.85**	**0.67**
		TP	FP	FN	TP	FP	FN	TP	FP	FN	TP	FP	FN	TP	FP	FN
P2Rank binding site filtering criterion	Fpocket (238)	172	66	112	240	472	75	186	100	99	246	514	71	318	3237	41
	SiteHound (167)	104	63	87	171	330	47	112	87	79	175	358	45	338	9866	7
	MetaPocket 2.0 (236)	186	50	90	249	459	52	194	90	84	253	503	52	311	1173	40
	DeepSite (238)	163	75	119	212	414	92	172	104	115	212	424	92	215	**546**	92
	P2Rank (238)	206	31	68	242	432	49	213	70	62	245	468	46	266	1296	41
	BiteNet (238)	213	25	**66**	283	**340**	38	**230**	**54**	**53**	290	**364**	32	315	734	27
	BiteNet R$^+$ (238)	**214**	**24**	**66**	**297**	411	**36**	229	57	55	**304**	452	**31**	**413**	2335	**17**
BiteNet binding site filtering criterion	Fpocket (238)	169	69	82	229	483	52	172	80	79	230	496	52	303	3252	20
	SiteHound (167)	103	64	75	169	332	35	108	70	68	173	339	32	325	9879	1
	MetaPocket 2.0 (236)	179	57	68	237	471	31	180	70	67	237	485	31	297	1187	20
	DeepSite (238)	158	80	93	202	424	69	160	91	93	202	426	69	206	**555**	68
	P2Rank (238)	203	34	46	238	436	28	205	45	44	240	446	27	262	1300	21
	BiteNet (238)	206	32	45	266	**357**	22	210	42	42	267	**366**	21	297	752	13
	BiteNet R$^+$ (238)	**208**	**30**	**42**	**283**	425	**17**	**211**	**41**	**40**	**286**	436	**14**	**393**	2355	**4**
BiteNet criterion for training	Fpocket (238)	91	147	161	111	601	141	92	160	160	111	615	141	132	3423	120
	SiteHound (167)	71	96	107	104	397	74	75	103	103	105	407	73	140	10064	38
	MetaPocket 2.0 (236)	99	137	150	124	584	126	99	151	150	124	598	126	147	1337	107
	DeepSite (238)	89	149	163	106	520	151	89	162	163	106	522	151	108	**653**	150
	P2Rank (238)	133	104	119	144	530	109	134	116	118	144	542	109	149	1413	104
	BiteNet (238)	176	62	75	196	**427**	55	177	75	74	196	**437**	55	202	847	49
	BiteNet R$^+$ (238)	**177**	**61**	**73**	**200**	508	**50**	**179**	**73**	**71**	**202**	520	**48**	**212**	2536	**38**

The best score values are denoted with bold font. Top TP, FP, FN scores apply only to methods assessed on the same set of proteins as BiteNet.

Supplementary Table 12: Performance metrics on the HOLO4K subset of 2848 proteins, which are not in the BiteNet training set and each structure contains at least one relevant binding site according to the BiteNet binding site filtering criterion.

Criterion	Method	Top-1			Top-3			Top-n			Top-(n+2)			All		
		prec	rec	ap	prec	rec	ap	prec	rec	ap	prec	rec	ap	prec	rec	ap
P2Rank binding site filtering criterion	Fpocket (2529)	0.65	0.30	0.20	0.38	0.53	0.33	0.49	0.54	0.34	0.32	0.64	0.38	0.07	0.79	0.40
	SiteHound (1826)	0.58	0.28	0.15	0.35	0.51	0.23	0.45	0.53	0.25	0.31	0.65	0.27	0.02	**0.97**	0.31
	MetaPocket 2.0 (1645)	0.70	0.41	0.26	0.37	0.64	0.41	0.55	0.61	0.40	0.33	0.72	0.44	0.21	0.76	0.45
	DeepSite (2516)	0.73	0.34	0.25	0.44	0.49	0.33	0.64	0.48	0.34	0.42	0.50	0.33	**0.38**	0.50	0.33
	P2Rank (2848)	0.85	0.42	0.34	0.47	0.66	0.54	0.63	0.71	0.59	0.37	0.76	0.60	0.13	0.80	0.60
	**BiteNet** (2848)	0.95	**0.43**	0.42	**0.59**	0.71	0.68	**0.79**	0.82	0.79	**0.50**	0.86	0.80	0.31	0.88	0.81
	**BiteNet R$^+$** (2848)	**0.95**	0.43	**0.42**	0.53	**0.72**	**0.69**	0.77	**0.83**	**0.80**	0.45	**0.87**	**0.81**	0.14	0.91	**0.82**
BiteNet binding site filtering criterion	Fpocket (2529)	0.59	0.33	0.20	0.34	0.59	0.33	0.51	0.53	0.31	0.31	0.68	0.37	0.06	0.85	0.39
	SiteHound (1826)	0.56	0.33	0.17	0.33	0.57	0.24	0.48	0.51	0.24	0.31	0.69	0.27	0.02	**1.00**	0.31
	MetaPocket 2.0 (1645)	0.67	0.47	0.29	0.35	0.73	0.44	0.61	0.64	0.40	0.33	0.79	0.46	0.19	0.84	0.47
	DeepSite (2516)	0.69	0.40	0.28	0.41	0.55	0.35	0.66	0.53	0.36	0.40	0.56	0.35	**0.35**	0.56	0.35
	P2Rank (2848)	0.83	0.48	0.38	0.44	0.75	0.57	0.73	0.77	0.61	0.39	0.83	0.63	0.12	0.88	0.63
	**BiteNet** (2848)	0.91	**0.51**	0.49	**0.54**	0.82	0.76	**0.85**	0.87	0.82	**0.49**	0.94	0.85	0.27	0.97	0.87
	**BiteNet R$^+$** (2848)	**0.91**	0.51	**0.49**	0.49	**0.83**	**0.78**	0.86	**0.88**	**0.85**	0.44	**0.95**	**0.87**	0.12	0.99	**0.88**
BiteNet criterion for training	Fpocket (2529)	0.32	0.18	0.05	0.19	0.31	0.09	0.28	0.28	0.08	0.17	0.36	0.10	0.03	0.47	0.11
	SiteHound (1826)	0.38	0.21	0.07	0.23	0.39	0.11	0.33	0.33	0.10	0.22	0.46	0.12	0.01	0.78	0.14
	MetaPocket 2.0 (1645)	0.38	0.25	0.08	0.20	0.40	0.12	0.33	0.33	0.10	0.19	0.44	0.13	0.12	0.50	0.14
	DeepSite (2516)	0.40	0.22	0.09	0.23	0.30	0.10	0.37	0.29	0.11	0.23	0.30	0.11	0.20	0.30	0.11
	P2Rank (2848)	0.52	0.29	0.12	0.27	0.44	0.17	0.45	0.45	0.18	0.24	0.49	0.19	0.08	0.52	0.19
	**BiteNet** (2848)	0.81	0.45	0.40	**0.48**	0.71	0.61	0.75	0.75	0.65	**0.43**	0.81	0.68	**0.24**	0.84	0.69
	**BiteNet R$^+$** (2848)	**0.81**	**0.45**	**0.41**	0.44	**0.73**	**0.65**	**0.76**	**0.77**	**0.69**	0.39	**0.83**	**0.72**	0.11	**0.86**	**0.72**
		TP	FP	FN	TP	FP	FN	TP	FP	FN	TP	FP	FN	TP	FP	FN
P2Rank binding site filtering criterion	Fpocket (2529)	1634	895	4231	3130	4442	2854	3016	2990	2792	3890	7141	2148	5819	61897	1247
	SiteHound (1826)	1055	771	3097	2216	3262	2112	2045	2265	2035	2935	5027	1488	7015	174397	110
	MetaPocket 2.0 (1645)	1145	500	1911	1953	2982	1152	1764	1343	1248	2290	3948	902	2661	7792	787
	DeepSite (2516)	1835	681	3940	2747	3299	3049	2559	1413	3115	2785	3509	3007	2800	4287	3001
	P2Rank (2848)	2428	420	3972	3975	4322	2302	4228	2456	1946	4666	7313	1643	5296	30274	1371
	BiteNet (2848)	2693	155	**3868**	4766	**2820**	1959	5374	**1130**	1241	6079	**4695**	981	6731	**11169**	839
	BiteNet R$^+$ (2848)	**2708**	**140**	3878	**4954**	3544	**1928**	**5530**	1233	**1158**	**6413**	5944	**886**	**8465**	31445	**613**
BiteNet binding site filtering criterion	Fpocket (2529)	1480	1049	3050	2793	4779	1897	2367	2219	2148	3256	6370	1477	5056	62660	671
	SiteHound (1826)	1015	811	2199	2069	3409	1394	1610	1649	1588	2490	4421	1021	6059	175353	2
	MetaPocket 2.0 (1645)	1104	541	1320	1841	3094	674	1509	954	896	2047	3654	526	2445	8008	400
	DeepSite (2516)	1740	776	2765	2562	3484	2040	2331	1205	2142	2595	3585	2005	2611	4476	1999
	P2Rank (2848)	2353	495	2685	3754	4543	1301	3783	1380	1196	4236	6340	862	4778	30792	645
	BiteNet (2848)	2584	264	**2517**	4418	**3168**	915	4452	697	655	5245	**4465**	302	5979	**11921**	151
	BiteNet R$^+$ (2848)	**2594**	**254**	2522	**4586**	3912	**898**	**4511**	**662**	**604**	**5457**	5358	**280**	**7513**	32397	**69**
BiteNet criterion for training	Fpocket (2529)	807	1722	3779	1426	6146	3157	1266	3326	3315	1660	7972	2921	2135	65581	2454
	SiteHound (1826)	686	1140	2572	1273	4205	1990	1079	2184	2177	1501	5414	1759	2547	178865	723
	MetaPocket 2.0 (1645)	619	1026	1857	981	3954	1497	815	1654	1660	1099	4608	1380	1255	9198	1249
	DeepSite (2516)	1010	1506	3561	1419	4627	3199	1310	2230	3262	1430	4751	3188	1435	5652	3185
	P2Rank (2848)	1480	1368	3688	2248	6049	2914	2326	2843	2830	2539	8043	2618	2678	32892	2485
	BiteNet (2848)	2301	547	2867	3671	**3915**	1492	3853	1301	1308	4170	**5543**	991	4311	**13589**	850
	BiteNet R$^+$ (2848)	**2316**	**532**	**2852**	**3772**	4726	**1391**	**3949**	**1230**	**1212**	**4258**	6563	**903**	**4440**	35470	**721**

The best score values are denoted with bold font. Top TP, FP, FN scores apply only to methods assessed on the same set of proteins as BiteNet.

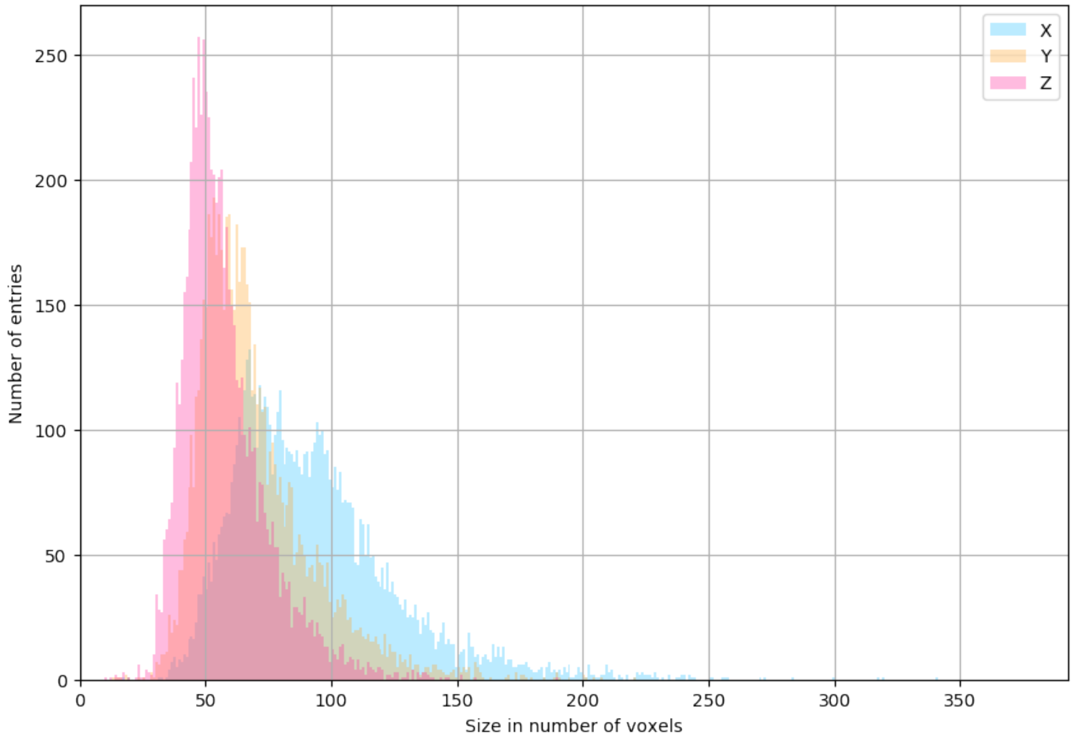

Supplementary Figure 5: Statistics for the protein lengths measured in voxels with respect to its principal axes.

Supplementary Table 13: The average number of binding sites in the COACH420 and HOLO4K benchmarks according to the P2Rank and BiteNet binding site filtering criteria (the first two rows), as well as the average number of method predictions for each dataset. For BiteNet the listed values correspond to the probability score thresholds of 0.01 and 0.1, respectively.

	COACH420	HOLO4K
P2Rank criterion	1.43	2.37
BiteNet criterion	1.05	1.84
Fpocket	14.60	27.01
SiteHound	60.23	99.87
MetaPocket 2.0	6.32	6.38
DeepSite	3.16	2.79
P2Rank	6.26	12.57
BiteNet	4.61/1.52	6.35/2.57

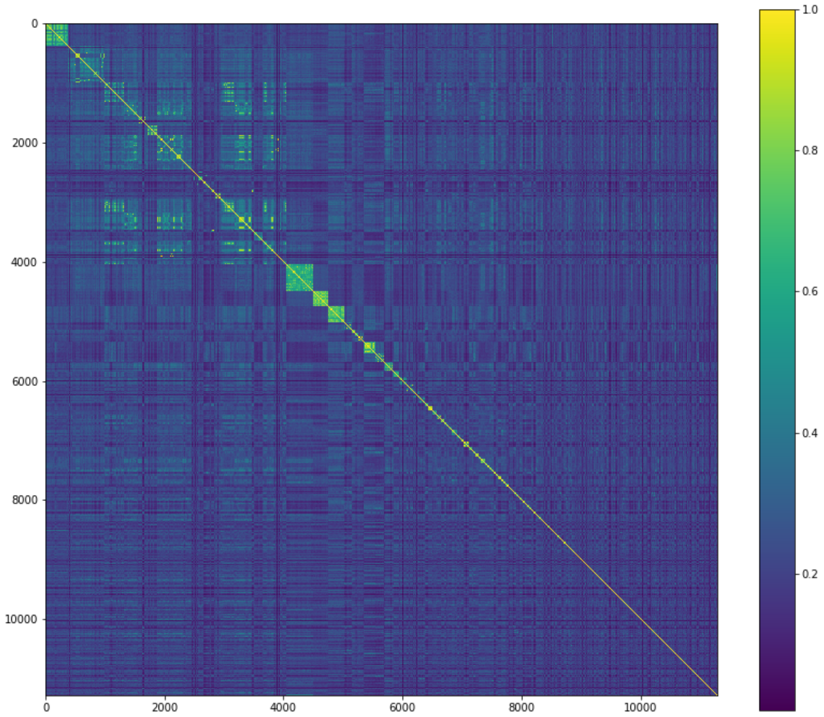

Supplementary Figure 6: Pair-wise structure similarity matrix calculated for the protein chains in the training dataset.

Supplementary Table 14: Statistical tests for comparison of performance of BiteNet with other methods on the set composed from COACH420 and HOLO4K subsets (238 and 1682 proteins, which are not in the BiteNet training set, predictions of all methods available and each structure contains at least one relevant binding site according to the in P2Rank binding site filtering criterion). The T-test is performed: the composed set was split into 31 independent subsets; for each subset precision, recall and average precision (AP) are calculated; the null hypothesis states that there is no significant difference in performance metrics between BiteNet and another method.

	Method	Precision		Recall		AP	
		t-statistic	p-value	t-statistic	p-value	t-statistic	p-value
Top-1	Fpocket	19.77	5.8e-28	10.77	1.2e-15	14.76	1.2e-21
	SiteHound	23.62	4.4e-32	12.53	2.1e-18	19.03	4.2e-27
	MetaPocket 2.0	21.45	8.0e-30	9.86	3.6e-14	16.69	3.3e-24
	DeepSite	17.40	4.1e-25	8.41	9.7e-12	13.17	2.3e-19
	P2Rank	7.90	7.4e-11	1.12	2.7e-01	5.41	1.2e-06
Top-3	Fpocket	17.84	1.2e-25	9.93	2.8e-14	16.96	1.4e-24
	SiteHound	16.43	7.0e-24	10.48	3.5e-15	22.30	1.0e-30
	MetaPocket 2.0	19.36	1.7e-27	10.18	1.1e-14	19.75	6.1e-28
	DeepSite	14.62	1.9e-21	13.85	2.4e-20	21.07	2.1e-29
	P2Rank	13.39	1.1e-19	2.95	4.6e-03	8.14	2.8e-11
Top-N	Fpocket	19.53	1.1e-27	13.05	3.5e-19	17.68	1.8e-25
	SiteHound	21.81	3.2e-30	14.77	1.2e-21	22.15	1.4e-30
	MetaPocket 2.0	21.39	9.1e-30	13.26	1.7e-19	20.55	7.7e-29
	DeepSite	14.11	1.0e-20	16.54	5.0e-24	20.83	3.7e-29
	P2Rank	13.87	2.3e-20	4.26	7.3e-05	8.39	1.1e-11
Top-(N+2)	Fpocket	19.76	6.0e-28	9.07	7.4e-13	17.22	6.9e-25
	SiteHound	16.73	2.9e-24	8.63	4.1e-12	22.39	7.9e-31
	MetaPocket 2.0	19.20	2.6e-27	9.55	1.2e-13	20.14	2.2e-28
	DeepSite	11.48	8.8e-17	18.08	5.8e-26	23.66	4.1e-32
	P2Rank	18.31	3.1e-26	3.75	4.0e-04	9.05	8.0e-13
All	Fpocket	42.87	1.0e-46	2.68	9.5e-03	17.19	7.4e-25
	SiteHound	52.85	5.0e-52	-13.02	4.0e-19	22.23	1.2e-30
	MetaPocket 2.0	18.32	3.0e-26	8.08	3.6e-11	20.35	1.3e-28
	DeepSite	1.18	2.4e-01	18.99	4.7e-27	24.34	8.8e-33
	P2Rank	30.67	2.3e-38	2.33	2.3e-02	9.56	1.1e-13
