# Supplementary material for: Spatiotemporal identification of druggable binding sites using deep learning
Source: Commun Biol. 2020 Oct 27;3:618. doi: 10.1038/s42003-020-01350-0 (PMC7591901; doi:10.1038/s42003-020-01350-0)
Supplement: Supplementary file 3 — Description of Additional Supplementary Files [file 42003_2020_1350_MOESM3_ESM.pdf]

Description of Additional Supplementary Files

File Name: Supplementary Movie 1
Description: **BiteNet applied to the minimization trajectory of the EGFR kinase domain starting from the unbound state.** Predictions corresponding to the orthosteric and allosteric sites are shown as yellow and magenta spheres, respectively.

File Name: Supplementary Movie 2
Description: **BiteNet applied to the ligand-free A2A molecular dynamics trajectory.** BiteNet predictions for the orthosteric and hypothethical binding sites are colored with yellow and magenta, respectively. Lipid molecule, that occupies the identified binding site, is shown with green sticks.

File Name: Supplementary Movie 3
Description: **BiteNet applied to the ligand-bound A2A molecular dynamics trajectory.** BiteNet predictions for the orthosteric and hypothethical binding sites are colored with yellow and magenta, respectively. Lipid molecule, that occupies the identified binding site, is shown with green sticks.

File Name: Supplementary Data 1
Description: Raw data for plots and tables
